# Exploring critical factors of the perceived usefulness of a learning analytics dashboard for distance university students

**DOI:** 10.1186/s41239-021-00284-9

**Published:** 2021-09-02

**Authors:** Irina Rets, Christothea Herodotou, Vaclav Bayer, Martin Hlosta, Bart Rienties

**Affiliations:** 1 Management School, Lancaster University, Lancaster, LA14YX UK; 2 Institute of Educational Technology, Open University UK, Milton Keynes, MK76AA UK; 3 Knowledge Media Institute, Open University UK, Milton Keynes, MK76AA UK

**Keywords:** Learning analytics dashboard, Technology acceptance, Perceived usefulness, Distance learning

## Abstract

Learning analytics dashboards (LADs) can provide learners with insights about their study progress through visualisations of the learner and learning data. Despite their potential usefulness to support learning, very few studies on LADs have considered learners’ needs and have engaged learners in the process of design and evaluation. Aligning with that, there is a limited understanding of what specific student cohorts, in particular distance and online learners, may seek from LADs to effectively support their studies. In this study, we present findings from 21 interviews with undergraduate distance learners, mainly high performers, that aimed to capture student perceptions about the usefulness of specific LAD features and the factors that explain these perceptions. Our findings revealed that amongst the LAD features favoured by students was the potential to receive study recommendations, whereas comparison with peers was amongst the least favoured elements, unless informed by qualitative information. Factors including information trust, attitudes, age, performance and academic self-confidence were found to explain these perceptions.

## Introduction

Assessment and physical attendance have traditionally played a central role in measuring students’ progress and level of engagement with their studies. These days, following a progressive move to blended and online learning, many universities are starting to collect and store other kinds of real-time evidence on students’ day-to-day learning and engagement, including, among others, their interaction with the course website, usage of the library and learning materials, past grades, timeliness of assignment submissions (Broughan & Prinsloo, 2020). Learning Analytics (LA), which provides means for collecting and analysing such evidence, has the potential to identify at-risk students and provide insights into how teaching and learning may be improved (Jones, 2019).

LA data are increasingly aggregated and presented to the end user in the form of a Learning Analytics Dashboard (LAD) (e.g., Matcha et al., 2019). Using real-time LA evidence, LADs provide a single focal point and a snapshot of students’ learning progress through one or more visualisations and learning recommendations (Sedrakyan et al., 2020). LADs are viewed as a promising way of facilitating self-regulated learning (e.g., Rienties et al., 2018; Schumacher & Ifenthaler, 2018), which refers to the students’ ability to set goals for their own learning, as well as monitor, control and adjust their behaviour to achieve them (Sedrakyan et al., 2020). Additional emerging evidence suggests that having access to a LAD has a positive impact on students’ grades, motivation to study and retention behaviour (de Quincey et al., 2019; Jivet et al., 2021).

Despite the promising potential of LADs to support students’ learning, these tools have been criticised for being too ‘quantified’ self focused, and lacking students’ feedback (de Quincey et al., 2019; Jivet et al., 2021; Zawacki-Richter et al., 2019). LADs are often designed for educators and university staff members as the target audience. Very few LADs are developed for use by students and informed by direct and comprehensive student evaluation, whose data are aggregated in the tool. A systematic literature review by Bodily and Verbert (2017) showed that out of 94 articles identified on student-facing LADs, only 6% included some form of student needs assessment.

Such a salient gap in research, that is analysing students’ reactions to LADs and their impact on learning, reflects more generally some of the inherent tensions that exist in the LA field. On the one hand, universities are committed to collect, measure, analyse and use student data to improve learning and act as custodians of academic standards. On the other hand, students, and the student voice, are often not part of the conversation (e.g., de Quincey et al., 2019; Zawacki-Richter et al., 2019). As Broughan and Prinsloo (2020) explained: ‘In the social imaginary of LA, students are habitually seen as the producers of data and as data-objects, but not as equals’ (p. 619). With the acknowledgement that students can become ‘objectified’ by LA, as well as with the increasing number of studies showing that technology used to support learning does not fit all (Rets et al., 2020; Rienties et al., 2020), there is a clear need to move towards a more student-centred approach to LA.

Exploring student voices in the context of distance learning is particularly important. Online distance education has seen huge increases in enrolments during the COVID-19 pandemic (Blackman, 2020), mainstreaming online teaching and learning across the different levels of education. Yet, this is not without challenges. Online teachers often rely on clues to assess whether their students are engaging and progressing, whereas distance learners studying at their own pace and time may or may not have the necessary skills to regulate their learning. LADs could be an important tool in the hands of teachers and students that can help them reflect on their learning, their learning progress, and possibly any support needed. Despite the current relevance of LADs and the fact that distance universities due to their size often have a greater capacity to collect larger amounts and quality of student data than traditional campus-based universities, as more learning interactions happen online, there are very few studies that have analysed distance learners’ response to and perceived usefulness of LADs.

Taking these two gaps into account—lack of LA research with the direct involvement of students in general, and distance learners in particular—this study details the perceptions of undergraduate students at a large distance university in the UK about the various elements of a LAD. It aims to investigate students’ perceived usefulness of the tool and what critical factors influence their responses.

## Literature review

### Learning analytics dashboards (LADs)

While the composition of individual LADs may vary, they are mainly designed around two common analytics approaches, namely descriptive and predictive analytics (Matcha et al., 2019; Schumacher & Ifenthaler, 2018; Schwendimann et al., 2016). Descriptive analytics deals with the digital footprints (also known as log data) learners leave behind whenever they interact with course materials and learning activities, and outlines information such as resource use, time spent online, progress towards the completion of a course. Predictive analytics provides predictions on course outcomes, such as the submission of the next assignment, based on students’ log data, historic data (e.g., past assignment/course grades) and socio-demographic data (e.g., previous qualifications). Besides outputting descriptive information and predictions, these two approaches enable LADs to provide peer comparison information and adaptive study recommendations. Although the latter is the least common element of LADs (Bodily & Verbert, 2017).

One crucial factor as to whether (or not) students would intend to use a LAD is their acceptance of this technology (Rienties et al., 2018). The Technology Acceptance Model (TAM), which predicts such intention is built on the constructs of the perceived usefulness (the extent to which a person believes that using a particular system would enhance their performance) and the perceived ease of use (the perceived effort it would take to use that system) (Davis, 1989). More recently, the TAM model has been extended to include other variables, such as trust, which refers to an individual’s willingness to be susceptible to a technology based on their expectations that the technology is predictable, reliable, and useful. Trust has been shown to have a substantial impact on the perceived usefulness and ease of use of technology (Zhu et al., 2020). In LAD research, it has been argued that students will only engage with a LAD if they trust the data and understand how they are calculated (de Quincey et al., 2019). Detailing the reasons behind why students receive a particular recommendation has been shown to increase trust in the system and the likelihood of them following the recommendation (e.g., Bodily & Verbert, 2017). A study by Klein et al. (2019) found that many students voiced concerns over control of their data, and their trust in how their institution handles the data, impacting the likelihood of their trust in the LAD (Klein et al., 2019).

Despite the fact that identifying the factors impacting perceived usefulness of LADs can help to support and enhance the effectiveness and efficiency of LADs, research on student-facing LADs with the direct involvement of students evaluating these tools is limited. Among the studies that started to address this gap is Schumacher and Ifenthaler (2018), who examined what elements of LADs were most sought after by university students. They found that students mostly welcomed the elements which support self-regulated learning, such as those that enable them to plan and organise their learning, provide personalised analyses of their learning and adaptive recommendations for study material. Furthermore, since 54% of participants in Schumacher and Ifenthaler (2018) indicated they preferred reading printed texts, the authors concluded that LADs should combine students’ offline learning activities with those online to improve the overall validity of how learning is presented. The need to see adaptive recommendations in LADs has been further highlighted in other recent studies. Sedrakyan et al. (2020) found that existing LAD instruments mostly provide outcome feedback (e.g., ‘How do I perform?’) rather than process-oriented feedback (‘How can I do better?’; e.g., by looking for inefficient processes in learning). The study questioned whether such feedback can be translated into a meaningful actionable recommendation to guide students in their learning.

Sedrakyan et al. (2020) accounted for the need to have adaptive recommendations in the LAD using the premises of the achievement goal theory. This theory distinguishes between mastery and performance orientations as the motivation behind why one engages in a learning task. In contrast to students with mastery goals, who are interested in learning as an end itself, students with performance goals are typically interested in learning as means of demonstrating their ability or competence (e.g., ‘I want to do better than other students in my class’) (Pintrich, 2000). In the achievement goal theory, mastery goals are associated with numerous desirable learning outcomes, as well as with the development of positive social relationships and moral development (Sedrakyan et al., 2020; Senko, 2016). Jivet et al., (2017) in their systematic review of LADs suggested that the design of current LADs is more appealing to performance-oriented students, neglecting the students who have a tendency towards mastery.

In contrast to the evidence that LADs can constrain students’ learning motivation in Sedrakyan et al. (2020) and Jivet et al. (2017), a different kind of evidence on the impact of a LAD on learning behaviour was obtained by de Quincey et al. (2019). Most students in this study acknowledged that having access to the LAD throughout the course made them reflect on a weekly/regular basis on their learning, instead of only working hard around assessment time. Furthermore, 80% of the students said that a decline in their weekly score influenced their behaviour in the following weeks. It incentivised them to improve their learning engagement and look around for missed content.

### Evaluation of LADs by different groups of students

Among the few studies on student-facing LADs, there are even fewer studies that analysed the impact of learner characteristics on student use and evaluation of these tools (Schwendimann et al., 2016). Emerging research evidence suggests that students’ academic achievement level and motivation play a role in their reaction to and use of the LAD. Kim et al. (2016) in their study with Korean students from a variety of majors, taking an online course on statistics, revealed a slightly positive correlation between student satisfaction with the LAD and their learning achievement, as measured by the final exam scores of the course. Kim et al. (2016) showed that high achieving students showed lower satisfaction with the LAD than the low achievers. The authors attributed this finding to the low-achieving students not knowing their learning status, compared to others, and, as a result, finding the LAD more motivating.

However, in a follow-up study with science and engineering students in a campus-based university in Belgium, Broos et al. (2017) found that the LAD was more appealing to ‘successful’ students. The authors found a noteworthy difference in the frequency of access of the LAD by the students at different academic achievement levels. More than half (56.3%) of the students with a very good study progress clicked through from the invitation to the LAD, while among the students with a low study progress, only 34.8% visited the LAD. The potential explanation the authors provided for this finding was that low-achieving students may have lost interest in obtaining additional information on their learning.

Besides the difference in the general satisfaction with the LAD among students, emerging evidence also suggests there might be a difference in students’ attitudes towards the peer comparison information presented in the LADs. Beheshitha et al. (2016) conducted a field experiment to examine the effect of LA visualizations on students’ participation in online discussions. The authors connected its findings to the achievement goal theory (Pintrich, 2000) and observed that students, who considered the subject matter of the course more motivating than competition between students were more inclined to rate negatively the LA visualisation based on social comparison. Roberts et al. (2017) used focus groups with Australian students working through three hypothetical scenarios of LADs. In contrast to Beheshitha et al. (2016), Roberts et al. (2017) found that students generally welcomed the feature to compare their performance relative to their peers. However, the students in the study expressed mixed messages about receiving alerts signifying their comparative performance.

Since most previous LAD studies (e.g., Beheshitha et al., 2016; Broos et al., 2017; Kim et al., 2016) were quantitative, it is important to verify this emerging and at times contradictory evidence by using qualitative methods. Furthermore, while these studies have been conducted within traditional campus-based university contexts, the challenges faced by distance learners may be more pertinent than the campus-based equivalent, where detection and interventions may be in place earlier (Rienties et al., 2018). Herodotou et al. (2020a) conducted a preliminary study with 19 middle-aged white distance learning students, who viewed the LAD of a hypothetical struggling student. The analysis of their answers to eight forum questions revealed a preference towards study recommendations among distance learners, while comparative peer data were viewed as demotivating and less useful. Given the current relevance of this context, particularly in the aftermath of the COVID-19 pandemic (Blackman, 2020), more research is required to examine distance learners’ perceived usefulness of LADs. As such, this study aims to answer the following research questions (RQs):

1. What are the most and least useful elements of the LAD as perceived by undergraduate distance university students?
2. What are the factors explaining the perceived usefulness of the LAD?

## Methodology

### Participants

Students enrolled at a business undergraduate course at the Open University (OU), the largest distance university in Europe, were invited to take part in the study. The study focused on this course in order to have some control over participants’ previous data literacy skills, as business students are more likely to understand the graphical and numerical data presented in a LAD. The course lasted 32 weeks, between February and September 2020. The students were expected to submit five Tutor Marked Assignments (TMAs) as part of the course in weeks 4, 8, 14, 22 and 32, with each TMA score weighting as 10; 15; 15; 30 and 30%. To pass the course, a student needed to get 40% of the overall weighted score, with more than 30% score gained for TMA 5. Each TMA consisted of several questions, which tested students’ acquired knowledge for the seven course blocks, the ability to select important information from the given text, develop arguments in the written text, use business language, cite the resources properly and communicate the ideas in the course discussion forums. The course had 2408 enrolled students with 14% passed with distinction, 30% passed, 34% withdrawn during the course and 21% completed the course, but did not meet the pass criteria. All their course materials and the discussion forum were accessible within the Virtual Learning Environment (VLE), the course platform hosting resources and activities associated with the course.

An email invitation to the study was sent out to 678 students registered on the course, whose contact details were shared with us by the research ethics committee. Twenty-one students agreed to take part, the response rate was 3.1%. Such response rate is similar to other studies with distance learners (e.g., Bolliger & Halupa, 2018). Nine students were male, and the mean age was 34.67 (SD = 11.06, min 19, max 54). Most participants had high school as their highest educational degree (*n* = 16), three had an undergraduate degree and two had a postgraduate degree. Such diversity in participant demographics is also representative of the student population in distance education institutions (Rizvi et al., 2019). The OU’s open access policy allows anyone to start an undergraduate degree irrespective of previous qualifications and expertise.

Participants in this study were half way through the course when the data were collected (Weeks 17–26). We obtained data on their final scores for the course: *n* = 14 passed with distinction (> 85 out of total 100 points), *n* = 6 passed (> 55 out of total 100 points), *n* = 1 failed the course. Thus, 67% of the sample passed the course with distinction. Although one participant in our sample failed the course, other low performing and at-risk students did not express interest to take part in this LAD research. As mentioned above, our enquiry into the pass rate of all students registered on the course revealed that only 14% of the whole class passed the course with distinction.

**Fig. 1:**
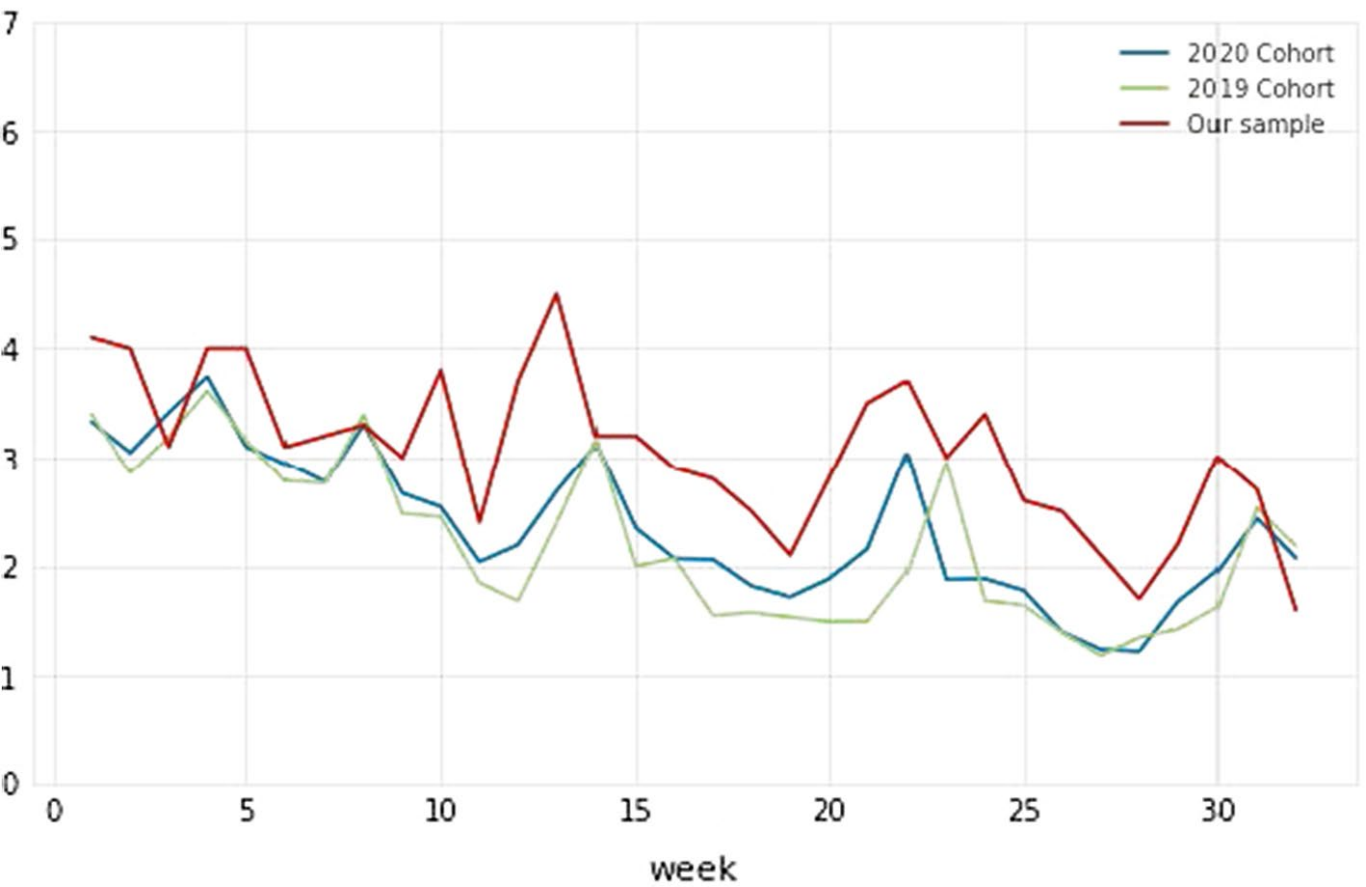
Average number of days per week for students that passed the course—our sample compared to the 2019 and 2020 cohorts for the course

Our exploration of participants’ log files further revealed that our sample was more engaged during the course than the rest of their cohort or the course cohort from the previous year. Figure 1 below visualises the average number of days per week that students were accessing VLE in three groups: (1) our sample compared with (2) 2020 cohort, i.e., the rest of that year’s cohort, and (3) 2019 cohort, i.e., the previous year. Since the sample mostly consisted of ‘passed’ and ‘distinction’ students, we restricted the graph only to this subgroup. It is widely reported in educational research that higher performing students tend to be more willing to participate in research, relative to students at-risk (Broos et al., 2017; Richardson, 2012).

### The LAD used in this study

**Fig. 2:**
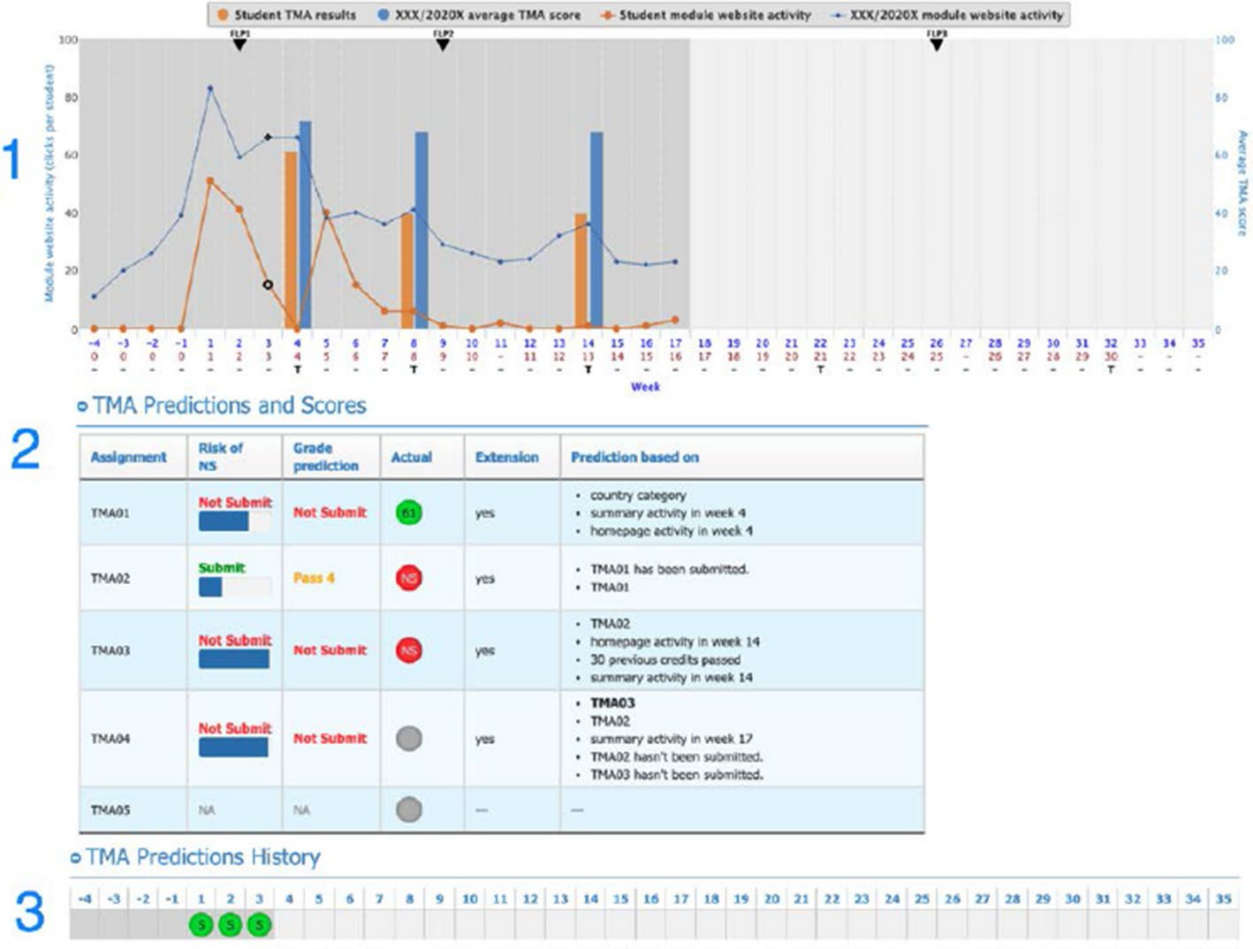
Student view of the LAD used in this study—VLE engagement and predictions

**Fig. 3:**
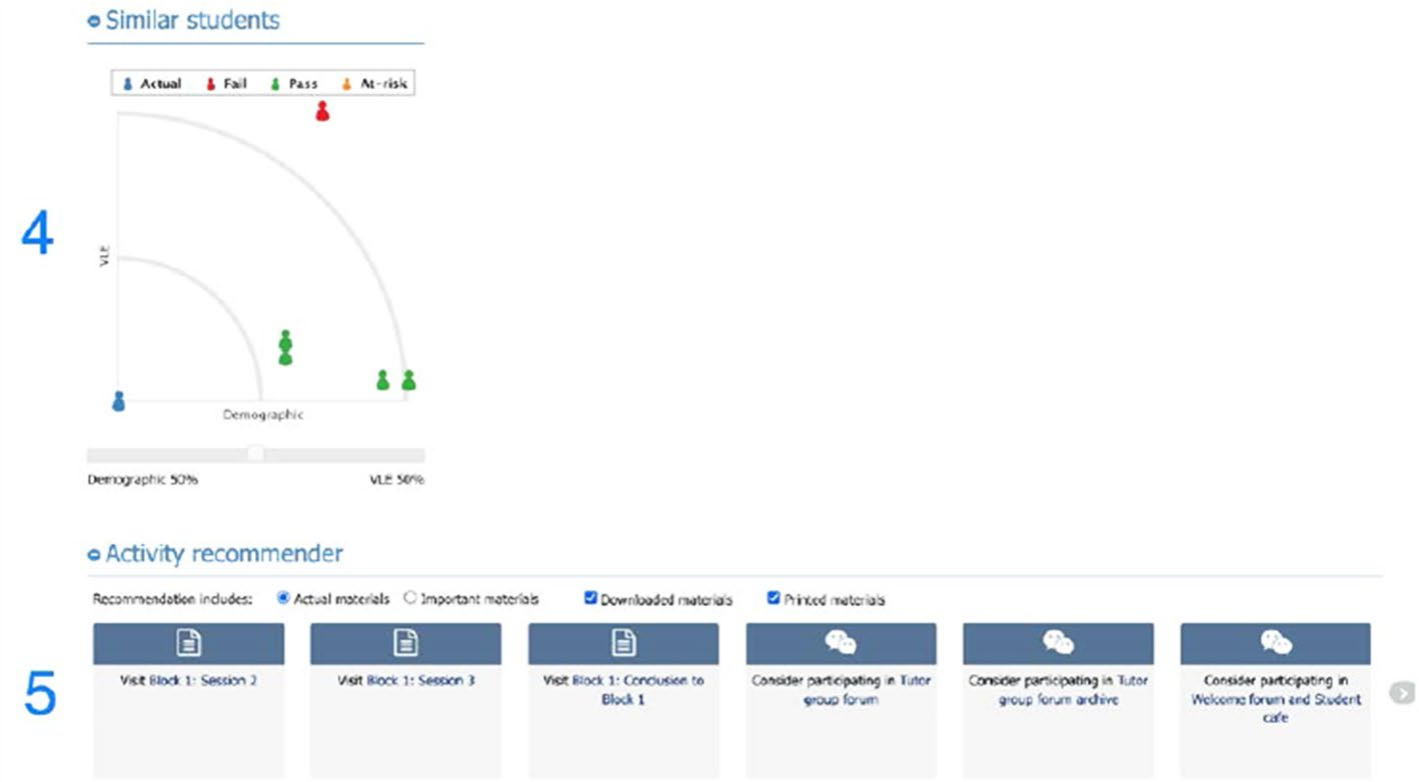
Student view of the LAD used in this study—similar students and study recommender

The LAD used in this study has been based on the OU Analyse predictive system and is an award-winning LAD. It is used across all faculties in the undergraduate modules of the OU and is currently only available to teachers. A detailed description of the LAD and the algorithm behind it can be found in our published work (Huptych et al., 2017; Hlosta et al., 2020). This study was the first attempt to share the LAD with the students and enable them to see and comment on their own LA data. While teachers at the OU have routine access to the LA data of both the whole course cohort and individual students, for privacy reasons participants in this study could only see a screen with their data. Specifically, they had access to the following features:

1. **VLE engagement graph** was an interactive graph showing weekly engagement in the VLE of a student compared with the average of the course cohort. Engagement was measured as a number of mouse clicks a student made during each week of the course. The graph also includes student’s TMA scores and the average score for the TMAs of the rest of the cohort.
2. **TMA predictions and scores** concerned the student’s past and future TMA submissions (S), the risk of non-submission (NS), and the potential banded grades (e.g., Pass 3—grade C, the scores between 55 and 69) they might receive. Predictions that are NS or Fail (score < 40) were shown as Red, the scores between 40 and 54 were shown as Amber and scores greater than 55—as Green. Each prediction was accompanied with the list of most significant attributes that contributed towards the generated prediction (e.g., low previous score or low activity in the weeks before the assignment cut-off).
3. **TMA predictions history** provided predictions generated each week of the course, whether the student was predicted to Submit (S) or Not Submit (NS) the next TMA.
4. **Similar students graph** displayed which students in the cohort were most similar to an individual student in the course based on their VLE activity and demographics. The displayed result could be adjusted by setting weights for the chosen criteria. Elements 2, 3, 4 used the traffic light system of colours, described in element 2 above. The blue coloured student in the left corner represented the student viewing the LAD.
5. **Study recommender** suggested sections of the course material that the student was encouraged to study next. These suggestions were based on the relevance of each material by taking into account the engagement of previously successful students (whose score was higher than 75%) with the course materials, combined with the individual student's engagement with them. For example, if two weeks before the TMA previous year’s successful students studied a material that was not mandatory as per the study plan, it would still have high relevance. Currently, the Study Recommender is still under development and is not part of the LAD version available for teachers at the OU. It has been included in this study, as previous research showed that adaptive learning recommendations constitute an important element for students (e.g., Herodotou et al., 2020a; Sedrakyan et al., 2020). The LAD used in this study, with elements from 1 to 5 described above, is visualised in Figs. 2 and 3 below.

### Procedure

The ethical clearance for this study was obtained from the research ethics committee of the OU. In line with previous studies that gave additional materials to participants, who do not have routine exposure to LA data (e.g., Schumacher & Ifenthaler, 2018), participants in this study were emailed a screenshot of a hypothetical student’s LAD to familiarise themselves with its design and clarify any comprehension questions.

Each research session took place remotely on Skype due to COVID-19 restrictions. The session started with a think-aloud interview. To prevent the loss of confidentiality resulting from participants accidentally viewing personal information of other students in their cohort, the interviewer had control when displaying the LAD to them. The interviewer shared their screen with the LAD with each participant, in which their own data from the course were displayed. As the interviewer was browsing the LAD, participants were asked to verbalise what they saw in the LAD, as well as to comment on their learning behaviours when interacting with the different LAD elements. In the second part of the session, the interviewer stopped sharing their screen and asked participants general questions about their experience of seeing the LAD and their LA data for the course (see Appendix for the list of questions). Each participant received a £25 Amazon voucher for their participation. The formulation of the retrospective interview questions was influenced by the TAM Model (Davis, 1989), and the questions were centred around the perceived usefulness of the LAD. Besides the interviewer, the interview with the first participant was attended by two other members of the research team, which was followed by a debrief and refinement of the interview questions.

The average length of the interviews was 45 min. Each interview was conducted in English, which was participants’ and interviewer’s first language, and was video and audio recorded during the Skype call using the functionality of the software. In this study, the names of participants are anonymised. All data from the 21 interviews were transcribed using automatic transcription service Otter (https://get.otter.ai/) and uploaded into NVivo11 for further analyses. The average length of the interview transcripts was 5000 words.

### Data analysis

To answer the study’s RQs, deductive (RQ1) and inductive (RQ2) thematic analyses were conducted of the think aloud and retrospective interviews in NVivo11 (Swain, 2018). The first author analysed and coded the data with one independent rater, compared and contrasted their codes with them, and then shared and discussed their codes with the rest of the team in a reflective session. Following an initial round of coding, the interview transcripts were reread for a critical assessment of assigned codes. The unit of analysis for coding was one paragraph (i.e., participants’ reflections on each element in the LAD during the think alouds and one full answer to a retrospective interview question). Responses could be given multiple codes. In light of the fact that previous research on student-facing LADs is limited, our thematic analysis as part of RQ1 followed the deductive approach and was informed by the TAM model (Davis, 1989). The analysis identified three codes—‘most useful’, ‘least useful’ element of the LAD, and ‘concerns over university surveillance’, which are directly described in Sect. 4.1. RQ2 in this study was more exploratory and, thus, was addressed using inductive thematic analysis. As part of RQ2, we identified 334 codes, which were divided into five themes (hereafter factors) that influenced participants’ perceived usefulness of the LAD: their attitudes towards *‘trustworthiness of the LAD’* and *‘peer comparison’*, the level of their *‘academic self-confidence’*, the extent to which the LAD would prompt a *‘change of study patterns’*, and participant’s *‘overall reaction to the LAD’* (see Table 1 below). We also linked the interview data from each participant to their demographic data, collected by the OU, including their age, gender, educational level, occupation type, as well as to their final scores for the course. In the instances where we identified mixed reactions to the LAD among participants, this information helped us understand what participant characteristics and experiences influenced their perceptions of the usefulness of the LAD.

**Table 1:** Final coding scheme with descriptions of themes (factors) of the perceived usefulness of the LAD

Theme	Description
Trustworthiness of the LAD	The extent to which participants believed the LAD provided an accurate representation of their study patterns; their reactions to off-track predictions and their appraisal of the mouse clicks
Peer comparison	The extent to which participants found peer comparison important and felt related to their peers
Academic self-confidence	The extent to which participants felt surprised or relieved after seeing the LAD
Change of study patterns	The kind of learning insights participants drew from the LAD; the extent to which they found the LAD motivating to study harder and monitor their learning progress
Overall reaction to the LAD	The extent to which participants either highlighted the advantages of the LAD or suggested its improvement

## Findings

### RQ1: most and least useful elements of the LAD

Given the limited research on student-facing LADs and the fact that this study’s participants did not have routine access to the tool, it was difficult to anticipate whether participants would voice any concerns over how or why the university uses their data. Our study showed that none of the participants spoke against university ‘surveillance’ or the collection of their personal data. Conversely, they often suggested that the LAD could collect additional data in the form of offline study, and personalised notes (see Sect. 4.2). One participant further noted that the proposed LAD is particularly relevant to the distance learning context, where students have limited contact with the teacher or peers: *‘It's nice to know at least that although it is distance learning, we're not completely unmonitored and left in the dark’ (P 9, female, 25, final score 80)*.

Despite such a uniformly positive attitude towards the collection and processing of student data, our analysis revealed that the perceived usefulness of the different elements of the LAD varied between participants (see Sect. 4.2). There were only two elements that were perceived similarly across the sample—the Study Recommender (element 5 in Fig. 3), which received mostly positive feedback, and the Similar Students graph (element 4 in Fig. 3), which consistently received negative reactions from participants. The majority of participants found the Study Recommender useful for two reasons: a) to remind them of the learning material they had missed, and b) as a means of directly accessing content (e.g., as opposed to going through the VLE). One participant further reflected that the Study Recommender can serve as a *‘pointer to think about how the student is engaging with the module website’ (P 13, female, 47, final score 88)*.

The consensus of the least useful element was the Similar Students graph. The majority of participants struggled to make sense of the semi-circular graph layout and required a detailed explanation from the interviewer. Many questions focused around the logic of plotting individual students on the graph and the displayed distance between them. Even after understanding the graph, participants noted that it would provide little benefit to them as ‘*even if those students are similar [to them], they might have a different way to organise themselves, a different way to study*’ *(P 18, male, 28, finals score 68)*. In essence, the Similar Students graph did not include the factors which participants considered important in relation to their studying, such as some insight into the profile of the students identified as similar to them, their approach to learning or their TMA scores. As one participant succinctly summarised: ‘*It could be useful to understand the detrimental factors that caused some students to fall behind. I would be looking at the reds and the oranges and sort of thinking, 'Why?' But you probably couldn't add that onto the diagram. (P 4, female, 25, final score 78)*.

While participants also interacted with the other elements of the LAD during interviews—the VLE Engagement graph (element 1), TMA Predictions and Scores (element 2), as well as TMA Predictions History (element 3), the perceived usefulness of these elements varied between participants. The following section details the factors that explain such a varied response. The enumeration of the LAD elements follows that in Figs. 2 and 3.

### RQ2: factors explaining the perceived usefulness of the proposed LAD

#### Trustworthiness of the LAD

The first factor revealed to have influenced participants’ perceived usefulness of the LAD was the level of appreciation and trust they displayed towards the LAD, and particularly towards elements 1 and 2. Some participants tended to question what they saw and frequently asked clarification questions about how these elements function. As the interviewer always provided the necessary clarifications upon request, at times participants disagreed with how these elements were designed. For example, some participants stated that student demographics should not be included into elements 1 and 2, as these factors are beyond the control of the student, in contrast to their engagement with the VLE.

P 13 (female, 47, final score 88): I'm from an ethnic minority. I'm an older student. I'm not from a particularly wealthy background. And if the algorithm took those factors into account and decided that my marks were going to be lower, because I have some inherent disadvantages, then that would be upsetting.

When interacting with element 3, some students were sensitive towards off-track predictions, the situations when the predicted grade about their past TMAs was lower than the actual grade.

P 13 (female, 47, final score 88): There was one [grade prediction], which was a Level 2. I feel a little bit undersold, quite frankly. It kind of makes me feel as though the university doesn't believe in my ability to succeed.

Another prominent sub-theme within the first factor was the idea that the number of clicks, which is taken into account in the LAD (particularly in element 1), does not represent quality of learning. Participants supported this idea by saying that a substantial number of clicks might involve navigation through the study materials or paying a casual visit to the course website, while being engaged in other activities, such as browsing social media or watching TV. Participants further explained that while the clicks indicate how active they were on the VLE, clicking does not reflect the extent to which they understood the material or the questions in the TMA. Three participants made comments that having a large number of clicks might indicate effort rather than learning: the student might be less tech-savvy.

P 8 (female, 36, final score 80): 185 clicks… you might even ask what I was busy clicking on. Is it like I got a stuck mouse or something?

P 10 (male, 54, final score 83): You could be active on the forum, the one that is part of the course, and you could post a lot of rubbish on there, as it still counts.

In contrast to the opinions described above on elements 1, 2 and 3, other participants tended to accept the information recorded in the LAD without probing it and made frequent comments about the accuracy of the displayed learning patterns. The accuracy they commented on particularly concerned their engagement (element 1). Quite a few participants were able to match the periods of low activity recorded in element 1 with the events happening in their private lives. Some participants also frequently commented on the fairness of the LAD and on the fact that it *‘creates a good baseline [for them] and what the system expects [of them]’* (P 4, female, 25).

P 14 (female, 21, final score 60): I do think it [the LAD] accurately represents how often I get on the module. Because I could see when I was on [the VLE] as much [as I could], my TMAs were slightly higher, and when I wasn't—my TMA results were slightly lower. I could also see, when it was low, what events were happening.

Thus, in relation to the first factor, some participants showed a much higher level of trustworthiness towards the LAD, than others. Having linked the demographics of participants with their interview data, our emerging evidence showed that older participants (M_age_ = 46.20, SD = 7.05) tended to trust the LAD less, while younger participants (M_age_ = 26.40, SD = 4.44) tended to talk more positively about its accuracy. In line with Pearce (2017), those participants who were above the age of 40, are, hereafter, referred to as ‘older’ or ‘more mature’ in this study.

#### Peer comparison

The second factor that influenced participants’ perceived influence of the LAD was how they positioned themselves in relation to their peers, as well as their attitude towards seeing the average score in element 1. Participants often mentioned in the interviews that they currently monitor their study performance by looking at the assignment scores they get throughout the course. Some participants found element 1 in the LAD not as useful and commented that they were aware from their scores that they were doing well and did not feel they needed to benchmark their performance against that of their peers.

P 1 (male, 43, final score 80): Like I said, I'm pretty aware that I'm probably an outlier in many respects. I wouldn't be looking for assurances from people being in the same catchment.

P 10 (male, 54, final score 83): Even though this is a Level 1 course, I still want to try and get what would be equivalent of the first [high grade]. It's more about comparing to myself rather than anything else.

In contrast, other participants found the peer comparison that element 1 enables important and liked the possibility of benchmarking their course activity and TMA scores with those of their classmates. The importance of peer comparison was particularly emphasised for distance learning, where students do not have close contact with their cohort.

P 21 (female, 33, final score 73): *If you're not in a classroom—so you don't know what other people are doing. And then you think, 'The grade that I've got—is it good, is it bad?' You don't get that because you're at home by yourself, and the only contact you have with people is through either the forum or when you're doing the tutorials.*

Having once again checked the demographic data of participants who provided such opposite opinions on the perceived usefulness of element 1, our emerging evidence showed a difference between ‘distinction’ and ‘pass’ participants. The former (M_final_score_ = 82.40, SD = 3.36) tended to be more sceptical about peer comparison than the latter (M_final_score_ = 69.20, SD = 6.76).

#### Academic self-confidence

The third differentiating factor closely connected to the factor above concerning participants’ attitude towards peer comparison was the level of participants’ academic self-confidence, or the extent to which participants felt surprised or relieved after seeing the LAD. Particularly in relation to elements 1 and 2 in the LAD, some participants often mentioned that the LAD confirmed what they already knew about their study progress. Seeing high predictions of their grades or high levels of activity in the VLE did not surprise them. Furthermore, some participants often talked about specific strategies they had to learning, such as having dedicated time regularly each week for studying. In relation to the latter, some participants wanted to see a personalised progress bar in the LAD that would somehow flag if *‘being too far ahead is counterproductive’* (P 10, male, 54, final score 83)*,* or whether this is a good learning strategy. Frequent mentioning of these strategies indicates that some participants in the sample were already able to self-regulate their learning well.

P 1 (male, 43, final score 80): Seeing the LAD has not changed really much in my opinion from how I think I'm doing. It is steady as it goes. I guess it's probably how I see my performance. Not setting the world on fire, but consistently, just above average, which is probably where I contend to be.

P 11 (male, 51, final score 81): I've now adopted this nearly daily activity. I do my reading in the morning. And then basically, in the evening. On weekends, I go on to the module website, or where I can I do at work. So, it’s just become like a daily habit.

In relation to this third factor, other participants, on the contrary, displayed low academic self-confidence. One of the most frequent reactions to the LAD among these participants was surprise that they were doing well and that they were above the average in their class, as well as relief. These participants once again emphasised the importance of having access to the LAD in the distance learning context. Since most of them study on a part-time basis and manage the conflicting demands of school, work and/or family, they talked about losing sight of how much effort they put into studying, which translates into not giving themselves enough credit. A few participants further mentioned having had anxiety before the interview for this study, as they could not anticipate what learning patterns they would see in the LAD. They then mentioned in the retrospective interview that participating in this research helped them feel better about themselves as learners and gave them confidence they would get a good final score for the course.

P 4 (female, 25, final score 78): It surprised me that I was doing above average, and because, like I say, I am my own biggest critic, I don't give myself enough credit. And sometimes I do think that everyone's probably doing better than me, and maybe I'm not doing as well as I could be. Seeing that I was above average—that really did surprise me in a fantastic way.

#### Change of study patterns

Consequently to the level of participants’ academic self-confidence and the extent to which they found the LAD informative, the fourth factor that influenced participants’ perceived influence of the LAD was their reflection on the extent to which the LAD would influence their approaches to learning. All participants indicated that seeing the LAD is good for sense-checking the amount of effort they put into studying. However, some participants often commented on seeing their performance in the LAD as not something that would encourage them to change these behaviours. These participants further commented that the LAD is self-intuitive—one does not need to see their activity levels to understand that the more effort they put into studying, the better result they get.

P 10 (male, 54, final score 83): I don’t think the LAD would enhance my study performance on this course, but I can see where it would, in the future. But, yeah, at the moment, I don't think it would, because the results I'm getting are probably pretty equivalent to what I normally would expect myself to get. I know what I'm doing in terms of putting time in etc.

On the contrary, other participants frequently mentioned that having access to the LAD would nudge them to reflect on and change their approaches to learning. Their comments on such a change concentrated around the opportunity to monitor their own learning progress and enhance their study performance. Participants noted that such monitoring enabled by the LAD would allow them to understand whether they are making progress and augment their study activity in line with the LAD predictions. These participants further suggested that the other way in which the LAD could influence a change in study patterns was through facilitating their motivation and self-confidence. This was related to monitoring, as students were able to see how well they were doing and whether they needed to put more effort in. An increase in motivation was usually noted as a result of participants noticing a drop in their VLE engagement levels in a given week in element 1 or a drop in their predicted grade in element 2. Consequently, a common point participants noted after seeing the LAD was to increase time spent in the VLE, as well as to study on a more regular or consistent basis, which would feed into their prediction scores.

P 4 (female, 25, final score 78): Those times where you start thinking, 'Right, I don't feel good about this TMA. I'm stressed, I'm anxious'. Just popping it [the LAD] on there and having a quick look would be the pep talk that you'd need. Or you could see, 'Well, actually, I haven't done that much. So really, I need to step up my game'.

Similarly to factor two above, for factors three and four we also found that the high-achieving ‘distinction’ participants, tended to be more sceptical towards the LAD (it confirmed what they thought about their progress and they did not feel encouraged to change their learning behaviours) and participants with lower final scores commented more positively about the LAD being informative for learning.

#### Overall reaction to the LAD

The final factor that influenced participants’ perceived influence of the LAD was their overall attitude to this tool and whether they emphasised the advantages of the LAD or whether they suggested areas for its improvement. The former was mainly voiced by the ‘pass’ participants (M_final_score_ = 69.20, SD = 6.76) and concerned the advantages of being able to monitor and enhance one’s study performance, described above. The ‘distinction’ participants hypothetically supported an idea of a student LAD. At the same time, they mainly suggested improvements that would help the LAD they were exposed to in the study provide more actionable insights.

The suggested improvements mainly concerned the need for greater personalisation of the LAD, overview of the global learning progress, the need to consider the offline learning when analysing student engagement, as well as the need for more study recommendations. Being able to input personalised data was a reoccurring point mentioned by several ‘distinction’ participants. They noted that a feature that would allow students to input their own grade expectations and enter notes in a comment box on their progress against their personal learning goals in element 2 would make the LAD more tailored towards individual student strengths. The interaction between the LAD and teachers was also mentioned, with several participants noting that being able to go through the LAD with their teacher would allow a contextualisation and better reflection on the data presented.

P 1 (male, 43, final score 80): If I was able to learn from the kind of feedback that I've got, so as I was going through a course, I was learning what my strengths or weaknesses were, and I could somehow input that—then the support becomes tailored to me.

These participants further mentioned that in its current form the LAD only provided an isolated view of their activity over one course and several suggested a LAD which displayed their global learning progress across several/all courses. As one participant put it: *‘I would really dearly love to see that dashboard in relation to my whole programme of study and not just to this module, so that I could get a sense of what type of a student I am, and maybe where I could be thinking of making some changes’ (P 13, female, 47, final score 88).* Participants believed that analytics for the entire study programme would enable them to compare their scores and identify *‘areas that are weaker’* or things they are *‘consistently just not getting’ (P 1, male, 43, final score 80)*.

Across the sample, some participants indicated that their preferred mode of study was using offline resources such as books or materials they had printed. As such, having a feature in which offline study could be recorded in the LAD was a widely mentioned recommendation. Being able to input the number of hours spent studying offline was noted to increase the accuracy of the predictive analytics, as *‘there’s [the] potential that you could be missing quite a lot in terms of the different [offline] study patterns’ (P 10, male, 54, final score 83).* Suggested methods to input offline study time included a diary/time log, or *‘another plot [to] log offline time’ (P 10, male, 54, final score 83)*.

As mentioned in 4.1., the great majority of participants felt positive about the Study Recommender. However, some participants indicated that they would like more prescriptive features like this, particularly the ‘distinction’ students. These participants wanted to get more direct advice on their learning, as compared to having a (predicted) overview of their learning activity. Suggestions for prescriptive features included materials students should focus on in a given week, textual feedback on how to achieve higher learning outcomes: *‘advice on how to get better would be useful, rather than just numbers’ (P 10, male, 54, final score 83)*, as well as how their results were directly impacted by interacting with different learning materials. Additionally, several participants noted they wanted a greater level of granularity in their study recommendations, including displaying an estimate of the reading time or a timeline of suggested learning activities.

The final reoccurring point mentioned in terms of the suggestions for LAD improvement was that the LAD required students to log on to view their progress and predicted grades. Participants noted that by not logging in regularly students may miss important changes in their predictions. To mitigate this, a frequently mentioned suggestion was that the LAD could send alerts in the form of emails, texts or through a mobile app. The perceived usefulness of alerts fell into two main categories: summarising activity over a weekly/monthly period and acting as prompts/warnings when students’ predictions were below average or their engagement with the course was below their usual performance:

P 13 (female, 47, final score 88): ‘What would be even more useful for me is to know exactly when I studied. So, what days, what times? So that I could be alerted when I don’t study during the times when I am most likely to study’.

While the suggested improvements fall into different categories, there seemed to be a shared motivation behind them—the idea of greater learner integrity. Participants, particularly who passed the course with distinction, wanted to get insights into the elements of effective learning—not only on the course for which they saw their data in the LAD, but also in terms of their long-term development as learners.

P 10 (male, 54, final score 83): I'm actually not targeting just to pass, or I am on this unit, because that's all there is. But you're targeting a grade. If you're looking for first class—2:1, or 2:2, it would be nice having those predictions on there, instead of just actual final pass for this course. Because hopefully I wouldn't be thinking about failing.

## Discussion

Despite an increased use of learning analytics dashboards (LADs) by higher education institutions, there is a lack of in-depth research on how students evaluate and respond to this tool. This gap in student-facing learning analytics (LA) research and the fact that the few studies on this topic have been conducted in traditional campus-based settings using mainly quantitative approaches, inspired this study. To that end, this qualitative study examined distance university students’ perceived usefulness of a LAD and investigated what critical factors influenced their responses.

As part of RQ1 we explored what element(s) of the LAD were perceived as most and least useful. Our study showed that the Study Recommender (element 5 in Fig. 3) was commended by all participants. Currently most LADs used by universities are outcome oriented (e.g., they help answer the question ‘How do I perform?’) (e.g., Sedrakyan et al., 2020). Our study supports the suggestions put forward by previous studies that LADs should provide more process-oriented feedback (e.g., ‘How can I do better?’) (e.g., Herodotou et al., 2020a; Schumacher & Ifenthaler, 2018; Sedrakyan et al., 2020).

Our study further showed that the Similar Students graph (element 4) was perceived as the least useful element of the LAD. While many participants still welcomed the social comparison functionality of the LAD in the VLE Engagement graph (element 1), our study demonstrated that in order for the social comparison to be informative for students, it needs to provide some information links between the individual student and the students they are comparing themselves with. Such information links may include other students’ exam scores, engagement with the course or insights into why they are behind or on top of their studying. This finding corroborates the assumptions behind the social comparison theory, which highlights the perceived closeness and relatedness with the peers as important attributes for social comparison (Festinger, 1954).

In contrast to the evidence from previous studies, that recorded students’ concerns over the control of their data, which impacted the likelihood of their trust in the LAD (Bodily & Verbert, 2017; de Quincey et al., 2019; Klein et al., 2019), our study revealed a uniformly positive attitude across the sample towards the university ‘surveilling’ their learning and designing the LAD based on the data it collects. On the one hand, this finding might be due to the fact that the OU was among the first higher education institutions to adopt clear ethical principles on the processing of student data, and, thus, our participants might have been more aware of the ethical implications in LA. However, participants in this study emphasised the usefulness of the LAD particularly in the distance learning context, where they do not have a chance to meet their cohort and benchmark their study progress, or where they can lose sight of their progress due to studying on a part-time basis. This finding constitutes an important insight, as, to our knowledge, there have been very few studies on student-facing LADs in distance education.

At the same time, our findings as part of RQ2 on the factors that explain the perceived usefulness of the LAD, showed that not all distance learners might benefit equally from the LAD. Their responses to the tool were influenced by the extent to which they trusted the information in the LAD, their attitudes towards peer comparison, the extent to which they found the LAD informative and motivating to reflect on their approaches to learning. Finally, their overall enjoyment of working with the LAD and whether they highlighted its advantages or suggested improvement were the substantial factors of influence.

Linking the interview data with the demographic and the academic achievement data available on participants revealed that in its current design, the LAD seemed more likely to appeal the most to younger students (< 40 years old) with low self-efficacy, who were successful in terms of their course pass rate, but who had medium-range scores.

These participants’ low self-efficacy manifested itself in the difficulties they had with evaluating their learning progress, apprehending their scores and more generally—understanding whether they are on track. The functionality of the LAD to pull information on their learning from many sources into one view and to benchmark their progress against that of their peers enhanced their academic self-confidence, while with some participants it reduced their anxieties about the scores for upcoming assignments.

This preliminary finding on the relationship between academic achievement, self-efficacy and the extent to which students find the LAD motivating supports the earlier study of Kim et al. (2016), who showed that high-achieving students showed lower satisfaction with the LAD than the low academic achievers. Furthermore, participants in our study, who commented that the LAD could prompt them to adjust their study patterns, attributed it to seeing a drop in their engagement or predicted grades. This finding is in line with de Quincey et al. (2019), where 80% of the students commented on a similar trend. In contrast to de Quincey et al. (2019) our analysis showed that such a response to the LAD is not uniform among students, and it is the students with low self-efficacy, who are more likely to be prompted by the LAD.

Our study further revealed that more mature students (> 40 years old) with high self-efficacy, who passed the course with distinction perceived the LAD as less useful. Besides commenting on the LAD not being very informative, these participants and particularly older students displayed low levels of appreciation and trust towards the LAD. They tended to probe and at times disagree with the design of the LAD and felt sensitive towards off-track score predictions. This finding is in line with previous studies that showed that people are generally less forgiving towards an algorithm than to humans, when the former makes a mistake, even when its overall performance is fine, with older people being less lenient (e.g., Araujo et al., 2020; Staddon, 2020). Since age has been shown to be negatively related to technology acceptance, it might explain the low levels of LAD appreciation among these students.

Our finding concerning mixed attitudes among students towards the usefulness of the peer comparison information presented in the LAD supports previous studies, which interpreted this finding from the position of the achievement goal theory (e.g., Beheshitha et al., 2016). It was beyond the scope of this study to record participants’ goal orientations. However, the analysis of participants’ comments about peer comparison suggests that many participants, who were sceptical about this element, might have had a tendency towards mastery (‘learning as an end itself), rather than performance goals (Pintrich, 2000; Sedrakyan et al., 2020). Although, previous research has shown that performance goals are not always maladaptive (Senko, 2016), our study supports the implications from Jivet et al. (2017) and Sedrakyan et al. (2020) in that the design of LADs should accommodate students with different goal orientation types. It should show awareness of the learning outcomes students target and support a student in both raising their social awareness and performing equally to peers (e.g., reaching the average score), as well as achieving the desired skills and competences.

Finally, our study showed that the improvements verbalised by participants that would increase the perceived usefulness of the LAD included the desire for greater personalisation and tracking of students’ progress against their own learning goals, tracking their progress across the curriculum and their learning with printed materials, providing more process-oriented feedback and alerting them if their performance falls below their usual levels. Some of these suggested improvements support the findings of the previous studies on the sought-after functionality of the LAD (e.g., Schumacher & Ifenthaler, 2018; Sedrakyan et al., 2020).

### Limitations

This study detailed the results of the first attempt of the university under study—the Open University (OU)—to expose its students to the LAD and enable them to see and comment on their own LA data. While this study provided emerging evidence on how different students perceive the usefulness of this tool and why, future research on the students, who have routine access to a LAD, might build a more naturalistic account of their perceptions of the tool. Furthermore, due to the small number of participants in this study, we treat the findings on the elicited differences in the perceived usefulness of the LAD between ‘distinction’ and ‘pass’ students, and between the more mature and younger students as emerging and exploratory. Future research in the form of a large-scale quantitative study should explore further the relation between demographic data and the preferences related to the LAD.

Secondly, our study showed that mainly successful students responded to take part in the study. This result corroborates the finding of Broos et al. (2017), who revealed that most students who clicked through from the email research invitation to the LAD were high-achieving students. This evidence is informative, considering that many efforts in LA are focused on at-risk students and drop-out prevention (Broos et al., 2017; Klein et al., 2019; Matcha et al., 2019). However, future studies should also explore how at-risk students interact with the LAD.

Finally, as this study was conducted at a large distance university, this raises issues of generalisability of the outcomes across other (campus-based) universities. At the same time, considering the current relevance of this context in the aftermath of the global pandemic and the fact that distance learning provides a unique context, where students with very diverse profiles (e.g., age) study together, the insights from this study have important implications for the design of LADs.

### Practical implications

One practical implication stems from the evidence that there were no ‘surveillance’ concerns about LA in this study and many participants wanted the LAD to be more personalised to them as learners. Thus, universities might consider collecting additional data about students in close consultation with them, such as the information about their offline learning, their personal learning goals, as well as psychometric data, such as the level of students’ self-efficacy and anxiety, which would increase the perceived usefulness of this tool. In the scenarios where such collection and storage of fine-grained personal data are not possible, it would be useful to incorporate the elements of a content management system into the LAD. With the latter functionality, the end user—the student—would be able to edit the LAD and personalise it, by, for example, logging in their offline study hours or setting up alerts and notifications about their study performance.

Secondly, the LAD could be used as part of teacher’s feedback on marked assignments to help students reflect on their learning progress. As our study showed, such reflection is important both for the students who feel they are behind and the students who are too far ahead with their learning material and it becomes counter-productive for their learning. This implication from our study concerning the use of the LAD as part of teachers’ feedback can also be supported by previous research. For example, Herodotou et al. (2020b) showed that the students, whose teachers used the LAD presented significantly better performance than their peers from the previous year, whose same respective teachers made no use of the LAD.

The third implication concerns the idea that the LAD should have the functionality to point students to additional information or clarification, written in an accessible, non-technical format, about how the different elements in the LAD are designed, why some predictions can be off-track, and why mouse clicks can be considered a good indication of students’ engagement and, thus, learning. Such clarifications have the potential to increase trustworthiness, and, thus, perceived usefulness of the LAD.

Finally, while the Study Recommender was perceived as most useful in this study, our study also showed that in order for the LAD to be useful for the more mature high-achieving students, it needs to provide more features, which would centre around the insights of effective learning and guide students in their development as life-long learners.

## Conclusions

This study illuminated the varied and different ways learners may perceive learning analytics dashboards (LADs) as well as the multiple factors that may explain their perceptions. These insights emphasise the need to include students and their voices throughout the process of designing LADs, as such tools should be personalised to the needs of specific students. Students should be seen as ‘equals’ (Broughan & Prinsloo, 2020) in the process of designing and refining LADs. Our previous work on designing LADs for teachers showed that the acceptance and adoption of such tools are heavily influenced by the engagement of end-users in all the stages of research (Rienties et al., 2018). A close collaboration with end users, in this study—students—in a participatory manner, would be beneficial in terms of designing LADs that respond and address specific students’ needs.

## Data Availability

The datasets used and/or analysed during the current study are available from the corresponding author on reasonable request.

## Appendix. List of questions used in the retrospective semi-structured interview

1. How did you feel when you saw the LAD?

2. Did any parts surprise you? If yes, what surprised you the most?

3. Do you find a LAD like the one you saw in this session a useful tool? If yes, in what way?

4. What element of the LAD did you find most useful? Least useful? And why?

5. To what extent do you think using a LAD would enhance your own study performance?

6. What might be the advantages and disadvantages of using a LAD in online learning?

7. If you could monitor your study performance online in any way, how would you prefer to do it?

8. Can using a LAD affect your student identity in any way, your feeling of self-worth?

9. What difficulties do you anticipate with using the LAD if it is integrated into the university courses?

10. If the university integrates a LAD in each course, will LAD fit well into your study routine?

## Abbreviations

LA: Learning analytics
LAD(s): Learning analytics dashboard(s)
NS: Non-submission
RQ(s): Research question(s)
S: Submissions
TMA(s): Tutor marked assignment(s)
VLE: Virtual Learning Environment
